# The Crosstalk between Cancer Stem Cells and Microenvironment Is Critical for Solid Tumor Progression: The Significant Contribution of Extracellular Vesicles

**DOI:** 10.1155/2018/6392198

**Published:** 2018-11-05

**Authors:** Chiara Ciardiello, Alessandra Leone, Alfredo Budillon

**Affiliations:** Experimental Pharmacology Unit, Istituto Nazionale Tumori—IRCCS—Fondazione G. Pascale, Naples, Italy

## Abstract

Several evidences nowadays demonstrated the critical role of the microenvironment in regulating cancer stem cells and their involvement in tumor progression. Extracellular vesicles (EVs) are considered as one of the most effective vehicles of information among cells. Accordingly, a number of studies led to the recognition of stem cell-associated EVs as new complexes able to contribute to cell fate determination of either normal or tumor cells. In this review, we aim to highlight an existing bidirectional role of EV-mediated communication—from cancer stem cells to microenvironment and also from microenvironment to cancer stem cells—in the most widespread solid cancers as prostate, breast, lung, and colon tumors.

## 1. Stemness: An Overview on Its Relevance in Cancer Development

Cancer stem cells (CSCs) represent a critical subset of the tumor population, which has been identified more than 10 years ago, able to promote cancer initiation and progression, contributing to therapy resistance, recurrence, and metastasis [1]. CSC theory of cancer progression described them as a specific compartment of tumor cells that, similar to normal stem cells, can induce hierarchical differentiation. CSCs showed ability to self-renewal, as well as invasive capability and metastatic proficiency, so favoring tumor aggressiveness [2, 3]. However, conflictive results have been obtained about either CSC origin or mechanisms by which CSCs serve as a critical tumor “tool” for resistance to anticancer therapy. Both an intrinsic therapy insensitivity belonging to nondividing CSC quiescent cells and resistance mechanisms activated by proliferative CSCs are hypotheses under debate. A key concept which unfolds cancer stem cell origin and dynamics in different malignancies is the “tumor plasticity,” providing the idea of dynamic changes affecting cancer cells, which explain both reversible mesenchymal transitions and acquisition of stemness traits, underlying the lethal biology of metastatic dissemination and development of resistance to treatments [2–5]. Hence, CSCs themselves do not exist as a static population, and the interconversion between CSCs and non-CSCs through self-differentiation and dedifferentiation has been proposed [6]. To date, the overexpression of few stemness-related transcriptional factors has been reported as able to transform non-CSCs into CSCs in both glioblastoma [7] and colon cancer [8] models. However, in the context of cancer, dynamic changes triggering tumor plasticity are (i) the conditions the tumor is usually exposed to (i.e., hypoxia) [9, 10]; (ii) the contribution of cell-to-cell communication exerted by EVs [11]; (iii) the tumor microenvironment (TME), composed of diverse cell types, such as mesenchymal stem cells, endothelial cells, fibroblasts, or immune cells [3, 12]. In this regard, Quante et al. demonstrated that bone-derived myofibroblasts favored the formation of a mesenchymal stem cell niche by a differential regulation of cytokines and secretory molecules such as IL6, Wnt5*α*, and BMP4, which ultimately leads to tumor progression and recurrence [13]. Concordantly, several studies demonstrated that cancer-associated fibroblasts (CAF), resident cells commonly present in the stroma, support the stemness of CSC cells by a paracrine mechanism. Indeed, it has been demonstrated that by the release of cancer cell-derived secretory molecules, CAFs could activate signaling functionally involved in the maintenance of stemness, as Wnt/*β*-catenin and Notch pathways [3, 14–17]. In return, as a feedback model, CSC influences CAF activity via activation of pathways functionally involved in cancer progression, such as Hedgehog signaling [18].

Beyond the cellular component, TME shows a *noncellular* component, defined as an extracellular matrix (ECM), which is composed of macromolecules such as collagens, glycoproteins, and proteoglycans as well as integrins [19, 20]. ECM, by both structure remodeling and a continuous crosstalk between tumor cells and the TME, regulates extracellular cues from the microenvironment in order to maintain CSC stemness or to promote differentiation into heterogeneous tumor phenotypes. Specifically, ECM molecules regulate CSC behaviors by modulating both cell-cell signaling and immune surveillance. For instance, tenascin-C, a protein of ECM involved in angiogenesis, invasion, and metastasis, has been recently identified as involved in the formation of the stem niche, relevant to favor lung colonization of breast cancer cells. Notably, this phenomenon seems to be dependent on the ability of tenascin-C to support the metastatic initiation of breast cancer cells through enhancing self-renewal pathways by increasing the expression of the regulator of stem cell signaling leucine-rich repeat containing G protein-coupled receptor 5 (LGR5) [3]. On the other hand, tenascin-C itself has been shown to induce immune escape of prostate stem-like cells, by disrupting T-cell activation [21]. Finally, tenascin-C seems to be correlated with poor prognosis in glioma patients, thus being also considered as putative CSC biomarkers for those patients [22, 23]. Both the survival of cancer cells and the formation of metastatic lesions have been recognized as deeply dependent on host microenvironment and specific organ structures, able to influence metastatic niche formation and interactions between cancer cells and local resident cells [24]. In this review, we aim to highlight an existing bidirectional role of EV-mediated communication—from cancer stem cells to microenvironment and also from microenvironment to cancer stem cells—in different solid tumors. In this context, we will describe how the CSC hypothesis provides an attractive cellular mechanism to account for the therapeutic refractoriness and dormant behavior exhibited by many solid tumors [25].

## 2. Extracellular Vesicles: Different Mediators Serving Cancer Development

Extracellular vesicles (EVs) are nowadays recognized as powerful mediators of intercellular communication in both physiological and pathological conditions. Their role in cancer development and progression has gained increasing attentions, in either hematologic or solid tumors, as broadly recapitulated in numerous reviews over the last years [26–30]. Tumor cells shed a heterogeneous set of EVs, and these spherical lipid bilayer vesicle populations differ in size, biogenesis, and molecular composition. Among the subtypes of EVs, the most studied are the *exosomes* (30–100 nm), which originate from the late endosomal trafficking machinery, gathered intracellularly into multivesicular bodies (MVBs) and shed upon MVB fusion with the plasma membrane [31]. In addition, *ectosomes*, *apoptotic bodies* (ABs), and *large oncosomes* (LO) represent additional subpopulations of EVs, which shared the feature to be secreted by budding from the cell plasma membrane (PM) and may express quantitatively and/or qualitatively different types of molecular components [32–35]. Actually, the ectosome category may be considered as inclusive of both ABs and possibly LO, which derive from apoptotic and nonapoptotic membrane blebbing processes, respectively. These two categories are both larger than 1 *μ*m, and LO may even reach 10 *μ*m, being also the unique population to be exclusively shed by cancer cells [36]. *Microvesicles* (MV) are small cell particles of heterogeneous size (100–1000 nm) and also PM-derived, which are extensively studied when derived from platelets and endothelial cells in relation to thrombotic disorders and diseases other than cancer [37].

One of the pioneering studies on EVs and cancer stemness was the one by Ratajczak and colleagues showing vesicle-mediated horizontal transfer of mRNA and protein from embryonic stem cells as critical for the maintenance of hematopoietic stem/progenitor cell stemness and pluripotency [38]. Several years later, Stik and colleagues published in 2017 a study on mesenchymal stromal cells releasing EVs able to modulate hematopoietic stem and progenitor cell gene expressions, maintaining their survival and clonogenic potential, presumably by preventing apoptosis [39].

**Figure 1:**
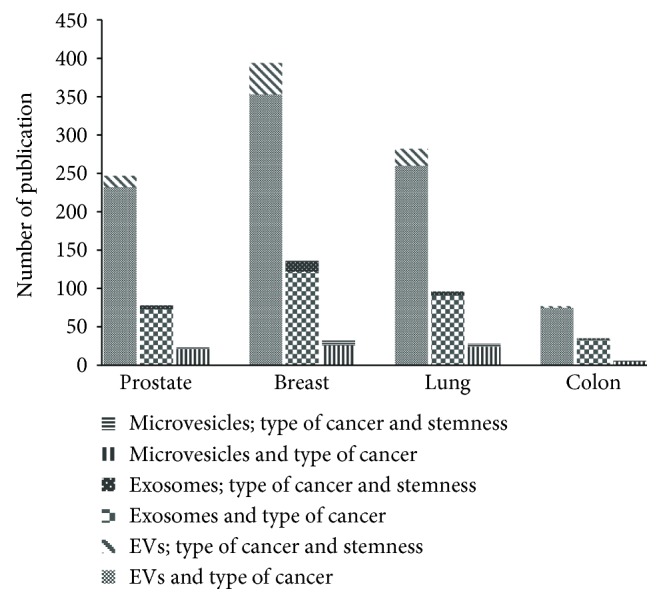
The graph shows the number of publication recorded in PubMed in the last 10 years (up to June 2018), by typing the following: 1st bar: extracellular vesicles “and” cancer (prostate, breast, lung, and colon) as well as extracellular vesicles “and” cancer “and” stemness; 2nd bar: exosomes “and” cancer (prostate, breast, lung, and colon) NOT extracellular vesicles as well as exosomes “and” cancer “and” stemness NOT extracellular vesicles; 3rd bar: microvesicles “and” cancer (prostate, breast, lung, and colon) NOT extracellular vesicles as well as microvesicles “and” cancer “and” stemness NOT extracellular vesicles.

Thus, it is not surprising to observe that in the last 10 years, a significant amount of studies have been focused on the correlation between EVs, cancer, and stemness, as highlighted by PubMed publications (Figure 1). However, an effort by researchers to uniform the nomenclature will help advances in the field enormously. As highlighted in Figure 1, different key words (EVs vs. exosomes,…) produced totally different outcomes despite the fact that each investigation aims to study the same topic.

## 3. Extracellular Vesicles from Cancer Stem Cells (CSCs) Influence Resident Tumor Cells and Tumor Microenvironment (TME) Carrying Different Molecules

The critical role of CSC in influencing TME has been highlighted and reviewed elsewhere [40]. The identification of new evolving communication factors in stem cell biology leads to the recognition of stem cell-associated EVs as new complexes able to contribute to cell fate determination of either normal or tumor cells. On one hand, SC-EVs could contribute to physiological activation of repair mechanisms after injury, by maintaining some key stemness features, such as self-renewal, differentiation, and maturation of damaged tissues [41]. In this regard, Tomasoni and colleagues unveiled how exosomes derived from bone marrow mesenchymal stem cells induced a horizontal transfer of insulin-like growth factor-1 mRNA, which ultimately support the repair of renal tubule after injury [42]. On the other hand, SC-EVs could influence tumor cell fate, by genetic reprogramming of resident cells and modification on TME as well as immunomodulation, which in turn could influence the tumorigenesis process [41]. Several studies have identified a number of miRNAs, such as miR-148a, miR-532-5p, miR-378, and let-7f, which, by regulating different genes involved in several multiorgan processes, could influence survival, differentiation, and immunomodulation of resident cells, including tumor cells [43–47]. In this regard, EVs derived from bone marrow mesenchymal stem cells delivered to tumor cells different miRNAs such as miR-23b and miR-21 and were able to sustain renal cell carcinoma and breast carcinoma proliferations, revealing a protumorigenic characteristic of MSC-EVs [46, 47]. Furthermore, EVs contain a large amount of proteins that could modulate several signaling pathways on resident cells. On instance, it has been demonstrated that EVs derived from mast cells acted as a shuttle for KIT proteins, which by activating its downstream pathway, leads to lung adenocarcinoma proliferation [41, 48]. Similarly, Roccaro and colleagues demonstrated that EVs from bone marrow mesenchymal stem cells transfer several cytokines, such as IL6, CCL2 (also known as MCP1), and junction plakoglobin (also known as *γ*-catenin), on melanoma cells, promoting tumor growth both on in vitro and in vivo models [49]. Finally, EVs carried also lipids, as diacylglycerol (DAG), sphingomyelin (SM), and ceramides, which are involved in the regulation of cell energy homeostasis as well as in crucial key pathways of tumorigenesis, such as proliferation, apoptosis, and migration [47, 50].

Some evidences lead to speculate that EVs, not only from stem cells but also from microenvironment, could promote, at least in part, the construction of premetastatic niches, by modulating the differentiation of the cellular component of TME [41, 51]. In this regard, it has been reported that gastric cancer exosomes induced differentiation of mesenchymal stem cells in CAFs, by transferring activation of molecules that ultimately modulate the TGF-*β*/Smad pathway [52]. In parallel, several evidences reported also a communication from cells of microenvironment on tumor cells by EV releasing, as recently reported by Shimoda et al. These authors showed that secretion of metalloproteinase-rich EVs from CAFs activates RhoA and Notch signalings, promoting cancer cell motility [53].

## 4. Extracellular Vesicles: Back and Forth Messages to Build a Network among the CSC Component and TME Cells, in Different Solid Tumors

### 4.1. Prostate Cancer

In the scenario of prostate cancer (PCa), studies available on CSCs (putative markers, localization within the organ, and functional studies) are still controversial, even if there are numerous evidences supporting the hierarchical model, in which a subpopulation of cells possesses the ability to initiate tumor growth and survival [54–56]. Recent studies on genetically engineered mouse models support the existence of cancer stem cells at diverse stages of tumor progression: from prostatic intraepithelial neoplasia to advanced metastatic and castration-resistant disease [57]. Maintaining CSCs in their undifferentiated stem cell state, which allows self-renewal and uninterrupted accumulation of genetic and epigenetic changes, is a condition triggered by several factors: one of the most studied in PCa is hypoxia [58]. From one hand, hypoxia has been identified as a promoting factor of metabolic changes, oncogene activation, and epithelial mesenchymal transition, resistant to chemo- and radiotherapy [59]; from the other hand, it has been also shown as able to affect EV-mediated communication [60]. Exosomes secreted by hypoxic cells were enriched in both HSP90 and HSP70 and expressed higher levels of annexin II compared to exosomes secreted by cells in normoxic conditions [61]. HSP90 has been described as abundantly secreted by organoids with cancer stem cell-like properties [62]; annexin II is also implicated in the metastatic process [63], and its expression in numerous cancers correlates with resistance to treatment, binding to the bone marrow, histological grade and type, TNM stage, and shortened overall survival, as discussed in a recent review [64]. Exosomes secreted by hypoxic condition-exposed PCa cell lines were able to either enhance the ability of naïve PCa cells to form *prostasphere or* promote the cancer-associated phenotype in prostate-associated CAF [61]. Indeed, exosomes have been shown to contain signaling molecules as TGF-*β*2, TNF1*α*, IL6, Akt, ILK1, and *β*-catenin primarily associated with the remodeling of the epithelial adherens junction pathway and stemness feature development [61].

**Figure 2:**
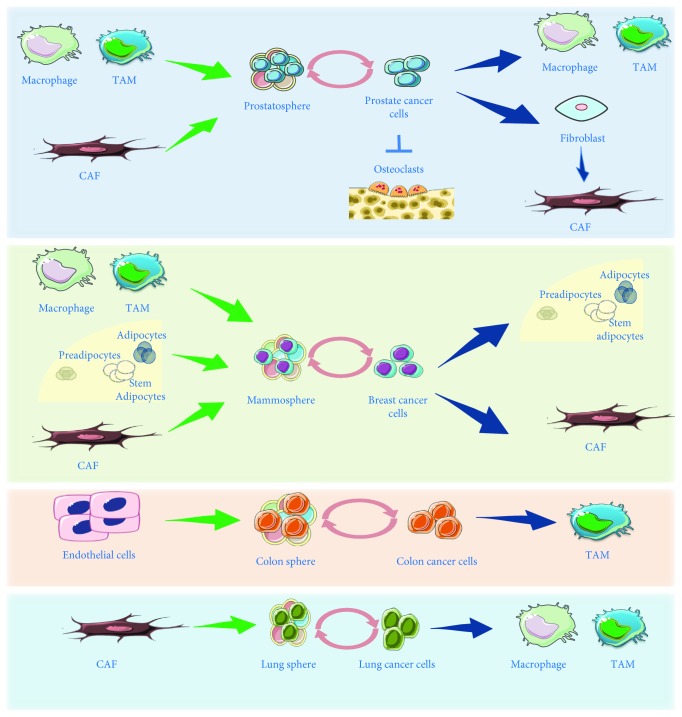
Specific interaction between microenvironment/metastatic niche components (i.e., fibroblasts, adipocytes, and macrophages) and organ-specific tumor cells through EVs, described in detail in the text. Tumor spheres are representative of a CSC-enriched tumor compartment, while tumor cells indicated a differentiated compartment. Green arrows indicated EV-mediated signals from microenvironment to tumor, blue arrows indicated EV-mediated signals from tumor to microenvironment, and light pink arrows indicated autocrine signaling mediated by EVs from tumor cells to the tumor sphere and vice versa.

The back and forth communication between PCa and TME to support stemness-related pathways is mostly studied referring to CAF, the key recipient of messages carried by EVs from PCa cell [65] macrophages and bone component (Figure 2). In detail, it has been recently shown that LO shed by PCa cell line LnCap^MyrAKT1^, harboring AKT1 kinase activity, were internalized by human normal prostate fibroblasts, inducing their reprogramming through the activation of stromal Myc [66], a proto-oncogene implicated in cancer initiation, maintenance, and stemness in different models [67, 68]. However, also miRNAs, carried by PCa CSC-derived EVs, have been shown to target fibroblast, affecting their proliferation, differentiation, and migration. Sánchez and colleagues compared exosomal miRNA shed from the “bulk component” versus CSC-enriched prostatosphere, both obtained from patient-derived primary cell cultures. They found hsa-miR-100-5p as the higher expressed in exosomes from both origin, compared to the other miRNAs [69]. miR-122 and let7b were both differentially expressed in CSC exosomes compared to bulk-derived exosomes [69], although their implications as stemness promoters are actually controversial [70, 71]. Some of the miRNAs coming out from that study are able to affect fibroblast properties as migration [69]. Conversely, it has been also reported that stromal fibroblast-derived miR-409 exported by EVs was able to induce activation of oncogenic, proliferative, EMT, and stemness programs of adjacent tumor epithelia *in vivo*; specifically, SOX2 and Nanog were both elevated in miR-409-expressing fibroblast [72]. An interesting study by Huang and colleagues unveiled the *reciprocal network* between CSC and macrophages, another major component of TME. In detail, the authors observed that the autophagy-related gene 7 (ATG7) facilitated the transcription of Oct4 via *β*-catenin, promoting CSC characteristics in prostate cancer, including self-renewal, tumor initiation, and drug resistance. In addition, also CSCs remodeled their specific niche by educating monocytes/macrophages towards tumor-associated macrophages (TAMs), and the CSC-educated TAMs reciprocally promoted the stem-like properties of CSCs as well as progression and ADT resistance of prostate cancer via interleukin 6 (IL6)/STAT3 [73]. Although in the latter study EVs were not mentioned at all, ILs exporting through EVs have been described [74].

Despite the fact that the bone is the preferential site of metastasis for breast and prostate tumors [75], only few studies explored the intercellular communication between PCa and both osteoblast and osteoclast. In detail, Karlsson and colleagues showed that exosomes isolated from the murine PCa cell line TRAMP-C1 dramatically decreased the fusion and differentiation of osteoclast precursors to mature multinucleated osteoclasts [76]. A clear decrease in the expression of established markers for osteoclast fusion and differentiation, including DC-STAMP, TRAP, cathepsin K, and MMP-9 was observed upon exposure to PCa-derived exosomes [76]. Inder and colleagues worked on EVs derived from the PC3 PCa cell line demonstrating that PC3-derived EVs were internalized by both osteoclast precursors and primary human osteoblast, inducing, respectively, osteoclastogenesis and proliferation [77].

### 4.2. Breast Cancer

Tumor initiation, therapeutic resistance, relapse, and metastasis have been associated to the concept of stemness and plasticity also in breast cancer [78]. It has been observed that *in vitro* models of breast cancer cells enriched in stemness features and grown as *mammosphere* showed a high expression of Rab27A (a member of RAS oncogene family), able to increase the exocytosis of EVs, compared to adherent breast cancer cell models [79]. Several studies focused on the effort to identify molecular cargos in EVs derived from breast cancer stem cells. miR-155 has been identified as enriched in exosomes isolated from breast CSCs, leading to EMT-associated chemoresistance [80]. miR-140, miR-29a, and miR-21 have been found to be enriched in exosomes derived from basal-like ductal carcinoma in situ (DCIS) stem-like cells. miR-9, upregulated in various breast cancer cell lines and identified as prometastatic miRNA, is delivered by exosomes and is able to affect the properties of human breast fibroblasts, enhancing the switch to CAF phenotype [81]. More recently, not only miR-9 but also miR-221 have been both shown to enable breast cancer cells to generate spheroids with stem cell-like characteristics [82]. A growing body of evidences reported EVs released from diverse cells belonging to the TME and targeting breast cancer stem cells. Among the variegated set of stromal cell types, numerous investigators have focused their work on bone marrow-derived cells, endothelial cells, fibroblasts, mesenchymal stem cell ability to influence tumor growth, and progression (Figure 2). In particular, on one hand, the ability of transformed fibroblasts to induce stemness markers in cancer cells (c-Myc/miR-34a circuitry deregulation, SOX2 upregulation,…) has been recently pointed out by Bono and colleagues [83]. In depth, miRNAs are often carried by exosomes as it has been nicely reviewed elsewhere [84]. Exosomes released by CAF have been shown to shuttle miR-21, -378e, and -143 and make breast cancer cell lines more efficient to form mammospheres, upregulating stemness-related transcriptional factors such as Oct3–4, Nanog, and SOX2 and promoting EMT via ZEB1 induction [85]. Already in 2003, other cell types belonging to TME gained attention as promoters of cancer aggressive features: adipocytes and adipose tissue-derived mesenchymal stem cells might contribute to a stem cell-like phenotype in breast cancer [86]. Despite the fact that this latest study by Iyengar and colleagues did not mention EVs, it suggested that “adipokines” were able to induce the expression of prooncogenic factors such as beta-catenin and CDK6 as a result of a reduction in the gene expression of their inhibitors in breast cancer recipient cells [86]. More recently, Baglio and colleagues set up a protocol for isolating exosomes released by both early passage adipose stem cell (ASC) and bone marrow MSCs (BMSC) and observed a selective export of miRNA in exosomes, not always reflecting the whole miRNA set of the cell of origin, thus suggesting a selective packaging process through EVs. miR-486-5p, miR-10a-5p, miR-10b-5p, miR-191-5p, and miR-222-3p were found to be the most abundant miRNAs in ASC exosomes, while miR-143-3p, miR-10b-5p, miR-486-5p, miR-22-3p, and miR-21-5p in BMSC exosomes [87]. Intriguingly, another study in 2015 showed that exosomes secreted from preadipocytes have been identified as important components of the cancer stem cell niche, significantly contributing, upon internalization by early-stage breast cancer cells, to mammosphere formation and growth [88]. On the opposite side, miR-503-3p, isolated from human adipose stem cell- (ASC-) derived exosomes, suppressed initiation and progression of CSCs, suppressing tumor sphere formation and decreasing the expression of pluripotency genes [89]. However, in order to highlight the reciprocity between breast cancer cells and adipocytes in communication through EVs, it is worth to mention that EVs shed by MDA MB-231 human breast cancer cells promote hallmark features of myofibroblastic differentiation and proangiogenic behavior in adipose-derived stem cells (ASCs) [90].

### 4.3. Lung Cancer

Lung cancer is one of the most common types of cancer, representing the leading cause of cancer-related deaths worldwide [91]. New lung cancer diagnoses increased 14%, and mortality related to lung cancer accounted for approximately 1 of every 4 cancer deaths in 2016 [91]. Two main histological subtypes of lung cancer have been described: small cell (SCLC) and NSCLC, the latter being the most frequent (close to 80–85% of all lung cancers) and aggressive (>5-year survival rate of 10%) [92]. Compared to other types of cancers, lung CSC markers have been poorly defined and explored since lung cancer is considered one of the most genotypic and histologically complex tumors [93]. Singh and colleagues showed that the signaling axis EGFR/Src/Akt is able to positively modulate SOX2 expression and self-renewal of stem-like side population cells in NSCLC [94]. More recently, NF-*κ*B inhibition has been shown to be sufficient to prevent the EMT and to induce apoptosis in lung CSCs, defined as CD166^+^CD44^+^, CD166^+^EpCAM^+^ cells [95]. Leprieur and colleagues described a Sonic Hedgehog (SH) membrane-bound full-length form, characterizing the CSCs compartment in human NSCLC, which has been observed to be secreted by CSCs, *in vitro* [96]*. In vivo*, analyzing 48 fresh human surgical samples, compared to healthy controls, the authors confirmed the *in vitro* observations suggesting paracrine and autocrine functions for SH protein, being responsible for CSC maintenance, tumor proliferation, and resistance to chemotherapy [96]. The CSC population in NSCLC has been recently defined also by the overexpression of the long noncoding DGCR5, which is able to regulate the expression of CD44 by modulating miR-330-5p [97]. The interplay and crosstalk between CSCs and TME, particularly CAF, have been shown to be relevant for lung cancer progression (Figure 2). Recently, Plou and colleagues demonstrated that modulation of collagen concentration and the amount of TGF-*β* in the microenvironment can regulate the plasticity of lung cancer cells, supporting the formation of tumor clusters, commonly considered enriched of putative tumor-initiating cells [98]. Furthermore, several studies demonstrated that CAFs promote lung tumorigenesis by activating a paracrine crosstalk with cancer cells and more importantly, with lung CSCs [99]. Indeed, Chen and colleagues identified the paracrine network by which the primary component of the NSCLC microenvironment, CAFs, enriches CSCs through dedifferentiation and reacquisition of stem cell-like properties. Specifically, they found that IGF1R signaling activation in cancer cells in the presence of CAFs expressing IGF-II can induce Nanog expression and promote stemness. Interestingly, the authors pointed out that this paracrine signaling predicts overall and relapse-free survival in stage I NSCLC patients [100].

Recently, several studies have highlighted the role of EVs containing CSC-priming factors as main agents to promote TME/CSC communication. To date, the literature available on EVs and lung cancer is mainly focused on the exosome population: a significant effort has been employed to define the exosomal miRNA content as diagnostic biomarkers for liquid biopsy, and, in parallel, EV-containing miRNAs have been demonstrated to play pleiotropic functions in regulating tumor malignancy [101]. A list of the main candidates has been published in a recent review [102]. However, what is immediately noticeable among these studies is either the variability in the sources of exosomes employed (mainly plasma and also sera or pleural effusions) or the higher variability in the methods to collect exosomes, thus affecting the reproducibility of these data sets.

A recent study by Hsu and colleagues observed that EVs shed by the human lung cancer cell line CL1-5 exposed to hypoxic conditions were enriched in miR-103a compared to EVs collected from the same cell line exposed to normoxic conditions [103]. They also showed that this EV-carried miR-103a was able to affect macrophage phenotype, inducing a tumor-promoting behavior via PTEN modulation [103]. Similarly, it has been reported that EVs such as exosomes secreted by H460 and A549 lung cancer cells modulate the tumor microenvironment, by influencing tumor cell migration. Mechanistically, the authors unveiled that restoration of LKB1, by targeting multiple critical signaling pathways, including AMPK/mTOR, p53, and PTEN/AKT, is able to inhibit exosomal secretion of migration-suppressing microRNAs (miRNAs), such as miR-125a, miR-126, and let7b [104].

### 4.4. Colon Cancer

Colorectal cancer (CRC) is nowadays considered as cause of approximately 10% of cancer-related mortality in western countries. As Kuipers and colleagues highlighted in 2015, diverse factors determined the rise in CRC incidence over the past 60 years, such as increasing ageing population, unfavorable modern dietary habits, and an increase in risk factors, such as smoking, low physical exercise, and obesity [105]. Feng and colleagues showed that, similar to other cancers and as mentioned above, Rab27A overexpression is correlated to increased sphere formation efficiency (SFE) and elevated secretion of VEGF and TGF-*β* from HT29 CRC cells [106]. They also found a correlation between a higher p65 level and Rab27A in the colon cancer sphere, demonstrating that NF-*κ*B signaling promotes various colon cancer stem cell properties via an amplified paracrine mechanism regulated by the higher Rab27A level [106]. To date, exosomes from CRC cells may export molecules in a selective manner, as mentioned above for other models: in detail, Cha and colleagues showed that the KRAS status of CRC cells can affect the type of miRNAs enriched in exosomes [107]. Considering the biological context in which CRC develops, it is worth to mention that there is a well-established link between CRC and chronic inflammation, which has been recently revised [108]. In detail, among the immune system cells, recent studies provide strong evidences that TAMs might facilitate CRC growth [109, 110]. Specifically, it has been shown that TAMs can stimulate CRC growth by altering extracellular matrix remodeling, tumor metabolism, angiogenesis, and the TME [110]. However, there are no studies focusing on the crosstalk between TAM and CRC; only a recent report unveiled that tumor-derived exosomes induce PD1^+^ macrophage population in human gastric cancer, promoting disease progression [111]. Exosomes from CRC have been indeed shown to directly induce the activation of mesenchymal stromal cells (MSC), isolated from colonic mucosa [112]. In detail, Lugini and colleagues showed that CRC exosomes are able to induce changes in the morphology of MSC accompanied by higher proliferation, migration, and invasion, formation of large 3D spheroids. In addition, they observed that colon cancer-derived MSCs, isolated from colon adenocarcinoma cell masses, fully recapitulate the changes observed in normal colonic MSCs exposed to CRC exosomes [112]. To support the TME in colon cancer, IL33, expressed by both cancer cells and endothelial cells, has been recently shown to stimulate CRC sphere formation and prevent chemotherapy-induced tumor apoptosis [113]. In detail, IL33 recruited macrophages into the cancer microenvironment and stimulated them to produce prostaglandin E2, which supported CRC stemness and tumor growth. In a recent study by Ren and colleagues, miR-196b-5p has been shown to promote either stemness or chemoresistance of CRC cells to 5-fluorouracil via targeting negative regulators of the STAT3 signaling pathway. Authors also found miR-196b-5p highly enriched in the serum exosomes of patients with CRC, compared to the healthy control subjects [114].

## 5. Conclusions

Although several pathways have been identified as mainly involved in maintaining a stem-supportive microenvironment in cancer development, the EVs role as mediators of stem signaling still needs to be deeply understood. In summary, what is known from recent literature is that on one hand, EVs derived from CSCs could influence tumor cell fate, by genetic reprogramming of resident cells and influencing the TME including immune cells (Figure 2 and Table 1); on the other hand, a specific component of the microenvironment (i.e., fibroblasts, adipocytes, and macrophages) may be able to modify the tumor niche by EV shedding in a diverse type of solid tumors (Figure 2 and Table 1). The characterization of the exact role of these different EVs and of their mRNA, miRNA, DNA, and/or protein cargos could help in the definition of novel tumor biomarkers as well as therapeutic targets.

## Figures and Tables

**Table 1:** Overview of biological interactions between tumor microenvironment and extracellular vesicles in solid cancer tumors.

Model	TME components	EV type	Cargo	Molecular targets	Ref.
Prostate cancer	NAF/CAF	LO	AKT	Myc activation	[66]
		Exosomes	miR-409	SOX and Nanog activation	[72]
	Macrophages	n.d.	ATG7 or IL6/STAT3	*β*-Catenin and Oct4 activation	[73]
	Osteoclasts	Exosomes	n.d.	DC-STAMP, TRAP, cathepsin K, and MMP9 inhibition	[76]

Breast cancer	NAF/CAF	Exosomes	miR-9	EFEMP1, COL1A1, and MMP1	[81]
			miR-21, -378e, and -143	Oct3/4, SOX2, and Nanog activation	[85]
	Preadipocytes	Exosomes	miR-149	SOX signaling	[88]

Lung cancer	Macrophages	Exosomes	miR-103a	PTEN modulation	[103]
	n.d.	n.d.	miR-125a, miR-126, and let7b	LKB1, AMPK/mTOR, p53, and PTEN/AKT	[104]

Colorectal cancer	Mesenchymal stromal cells	Exosomes	n.d.	Vacuolar H^+^-ATPase protein	[112]
	Macrophages	n.d.	n.d.	IL33/prostaglandin E2 signaling	[113]
	n.d.	Exosomes	miR-196b-5p	STAT3 signaling	[114]
